# Finding positive SARS-CoV-2 RT-PCR in cerebrospinal fluid of two pediatric patients with severe COVID-19: a brief case report

**DOI:** 10.1186/s12887-022-03806-0

**Published:** 2023-01-30

**Authors:** Reza Sinaei, Habibeh Nejadbiglari, Roya Sinaei, Maziar Zeinaly, Sara Pezeshki, Maedeh Jafari

**Affiliations:** 1 Department of Pediatrics, School of Medicine, Kerman University of Medical Sciences, Kerman, Iran; 2 Endocrinology and Metabolism Research Center, Institute of Basic and Clinical Physiology Sciences, Kerman University of Medical Sciences, Kerman, Iran; 3 Neuroscience Research Center, Institute of Neuropharmacology, Kerman University of Medical Sciences, Kerman, Iran; 4 Department of Pediatric Neurology, Pediatrics Center of Excellence, Children’s Medical Center, Tehran University of Medical Sciences, Tehran, Iran; 5 Department of Pediatrics, Kerman University of Medical Sciences, Kerman, Iran; 6 Department of Pediatrics, School of Medicine, Kerman University of Medical Sciences, Kerman, Iran; 7 Department of Internal Medicine, Kerman University of Medical Sciences, Kerman, Iran; 8 Endocrinology and Metabolism Research Center, Institute of Basic and Clinical Physiology Sciences, Kerman University of Medical Sciences, Kerman, Iran; 9 Clinical Research Development Unit, Afzalipour Hospital, Kerman University of Medical Sciences, Kerman, Iran; 10 Department of Pediatrics, School of Medicine, Kerman University of Medical Sciences, Kerman, Iran

**Keywords:** Covid-19, SARS-CoV-2, Cerebrospinal fluid, Neurotropism, Pediatric

## Abstract

**Background:**

There is growing evidence of nervous system involvement and related complaints in children with coronavirus disease 2019 (COVID-19). However, it seems that attempts to track of the virus in the nervous system have so far been unsuccessful.

**Case presentation:**

Here we describe two pediatric cases of severe COVID-19 who had positive cerebrospinal fluid (CSF) and nasopharyngeal polymerase chain reaction (PCR) tests for severe acute respiratory syndrome coronavirus disease 2019 (SARS-CoV-2). A 36-month-old girl who presented with fever, diarrhea, mild left ventricular dysfunction and bizarre movements, and a five-month-old boy who presented with fever, watery diarrhea, severe dehydration, mottling, and two episodes of seizure. Their CSF analyses and cultures were normal. They admitted in intensive care unit (ICU) for near four days and discharged after ten days without any complaint.

**Conclusion:**

This is one of the first reports of the presence of coronavirus in the central nervous system in COVID-19 pediatric patients, emphasizing the neurotropism and neuroinvasion characteristics of the virus.

## Background

There are increasing reports of the occurrence of both cases of multi-systemic [1, 2], and sporadic organ system involvements in the absence of definite hyper-inflammatory situations [3, 4]. Surprisingly, conflicts are being reported in both the central and peripheral nervous systems (CNS and PNS). The proposed mechanisms of CNS involvement include retrograde entry from the olfactory nerve, entry into CNS via circulating lymphocytes and via permeable blood brain barrier [5].

What enhances the uniqueness of this report is few pediatric reports, representing an objective evidence of the virus footprint in the CSF of COVID-19 affected patients.

## Case presentation

### Case-1

A three-year-old healthy girl hospitalized at the pediatric ward of Afzalipour hospital, Kerman, in the Southeast of Iran, due to fever and watery diarrhea from two-days ago. She experienced an episode of seizure for five minute just before the time of hospitalization. The patient transferred to pediatric ICU (PICU) due to his general condition including moderate to severe dehydration, drowsiness, tachycardia and oliguria. At the time of admission, her vital signs including temperature (T), respiratory rate (RR), pulse rate (PR), and blood pressure were 38˚C, 25 beats/min, 120 beats/min, and 80/50 mm Hg, respectively. The oxygen saturation was 95% in room air without additional oxygen.

Laboratory evaluation revealed a white blood cell (WBC) count of 12 $\times$ 10^9^ /L with an Absolute Neutrophil Count (ANC) of 9.6 $\times$ 10^9^ /L and an Absolute Lymphocyte Count (ALC) of $2.1\times$ 10^9^ /L, without any atypical lymph and blast cells. The platelet count was 222,000/µL, and the hemoglobin level was 12.6 g/dl. The Erythrocyte Sediment Rate (ESR) and C-reactive protein (CRP) were 47 mm/h and 60 mg/L, respectively. The Brain natriuretic peptide (BNP) and d-Dimer values were elevated. The patient underwent lumbar puncture, and the CSF was clear without evidence of bacteria in the smear and the cells in the analysis (Table 1). In abdominal ultrasound, both kidneys had normal sizes and the parenchymal echo of both side were increased. The chest X-Ray (CXR) showed no obvious abnormality. The brain CT-Scan showed only evidence of minimal brain edema. The echocardiography showed moderate mitral and tricuspid regurgitation in the presence of diminished ejection fraction to 43%, suggestive of left ventricular dysfunction. Considering the precede symptoms, the SARS-CoV-2 PCR from nasopharyngeal and CSF samples done, with positive results. Viral RNA was extracted using an automated nucleic acid isolation system (Zybio, EXM6000) according to manufacture manual and the product was processed afterthat. Detection of SARS-CoV-2 by one stage real time-PCR (RT-PCR) was performed, using the current kits used in Iran (http://pishtazteb.com/en/products/molecular-kits/covid-19-one-step-rt-pcr) according to the manufacture’s protocol [6]. Unfortunately, the Magnetic resonance imaging (MRI) as complementary facility was not performed. Treatment was planned by multidisciplinary consultation of team members of pediatric subspecialists. The patient underwent resuscitation by sufficient doses of normal saline, Lasix, epinephrine, dobutamin, phenytoin and other preliminary therapeutic strategies. A wide-spectrum coverage of bacteria was performed, administrating the ceftriaxone (80 mg/kg/day) and clindamycin (40 mg/kg/day). Subsequently, intravenous immunoglobulin (IVIG) and intravenous methylprednisolone prescribed at the doses of 2 g/kg and 2 mg/kg/day, respectively. The bizarre movements and the state of fear in the absence of encephalitis were interrupted with haloperidol, successfully. The patient survived near four-days in PICU and afterthat six-days in rheumatology service. She completely recovered clinically within 10-days and discharged with good condition. She had no neurological sign and symptom at the time of discharge. She followed at least for three-months later, without any subsequent sign or sequel.

**Table 1:** The Laboratory findings at the time of admission

Laboratory Test	Case-1 (3-year-old girl)	Case-2 (5-month-old boy)
WBC^a^ (5–14.5) × 10^3/µL	12	12.9
RBC^b^ (3.9–5.3) × 10^6/ µL	5.8	4.11
ANC^c^	9.6	9.69
ALC^d^	2.1	$2.65$
Hemoglobin (11.5–15.5 g/dL)	12.6	12.2
MCV^e^(75–87 fl)	86.4	82
Platelet(172–450)10^3/ µL	222	406
ESR^f^(0–15 mm/h)	47	25
CRP^g^(0–10 mg/l)	60	15
Blood smear for blast	Negative	Negative
BUN^h^(5–18 mg/dL)	12	23
Creatinine (0.5–1 mg/dL)	0.4	0.3
AST^i^(8–33 IU/L)	31	23
ALT^j^(10–40 IU/L)	12	12
Alkaline Phosphatase (180–1200 IU/mL)	344	339
PTH-i^k^(15–65 pg/mL)	41.0	NA
Ionized Ca (4.6–5.28 mg/dL)	3.7	4.5
Albumin (3.4–5.4 g/dL)	2.7	3.7
Ferritin (11–92 ng/mL)	NA^p^	NA
LDH^l^(< 746 U/L)	825	NA
d-Dimer (< 250 ng/ml)	400	NA
Pro-BNP^m^(< 125 pg/ml)	10,000	NA
Procalcitonin (< 0.5)	0.96	NA
Lupus anticoagulant (46–51)	30	NA
Anti-beta-2 Glycoprotein IgM (< 12)	2.2	NA
Anti-beta-2 Glycoprotein IgG (< 12	2.5	NA
Anti-cardiolipin IgM (< 15)	3	NA
Anti-cardiolipin IgG (< 15)	5	NA
Stool culture	Negative	Negative
Urine culture	Negative	Negative
CSF^n^ analysis	Color = clear and colorless Glucose = 43, Protein = 46, cell = negative	Color = clear and colorless Glucose = 73, Protein = 24, cell = negative
CSF culture	Negative	Negative
Coronavirus IgM (< 1.1)	NA	NA
Coronavirus IgG (< 1.1)	NA	NA
VCA^o^IgM	Negative	Negative
SARSA-CoV-2 nasopharyngeal PCR	+	+
SARS-CoV-2 CSF PCR	+	+

^a^*WBC* White blood cell count

^b^*RBC* Red blood cell count

^c^*ANC* Absolute neutrophil count

^d^*ALC* Absolute lymphocyte count

^e^*MCV* Mean corpuscular volume

^f^*ESR* Erythrocyte sedimentation rate

^g^*CRP* C-reactive protein

^h^*BUN* Blood urea nitrogen

^i^*AST* Aspartate aminotransferase

^j^*ALT* Alanine aminotransferase

^k^*PTH*-i intact parathyroid hormone

^l^*LDH* Lactate dehydrogenase

^m^*BNP* Brain natriuretic peptide

^n^*CSF* Cerebral spinal fluid

^o^*VCA* Varicella capsid antigen

^p^*NA* Not assessed

### Case-2

A five-month-old boy who had high-grade fever for two-days and watery diarrhea, admitted at Afzalipour hospital, Kerman. The patient transferred to PICU due to severe dehydration, mottling, acrohypothermia, and tachycardia. His T, PR, RR, and blood pressure were 39˚C, 114 beats/min, 35 beats/min, and 65/40 mm Hg, respectively. The O2 saturation was 85% in room air. The patient experienced two episodes of seizure, while the second was longer than thirty minutes as ‟status epilepticus”. Laboratory evaluation revealed a WBC count of 12.9 $\times$ 10^9^ /L with an ALC of $2.65\times$ 10^9^ /L, without evidence of blast cells. The ESR and CRP were 25 mm/h and 15 mg/L, respectively. All fluid cultures including blood, urine and CSF were negative. The nasopharyngeal and CSF swabs for coronavirus PCR assay were positive. The CXR showed mild patchy infiltrates in both sides. The echocardiography and the brain CT-Scan in the lack of MRI showed no obvious abnormality. The patient underwent resuscitation by intravenous hydration, dobutamin, phenobarbital (20 mg/kg as loading dose and 5 mg/kg/day as maintenance therapy), levetiracetam (15 mg q12-hours), remdesivir (15 mg/day for 5-days), meropenem (20 mg/kg q8-hours) and clindamycin (10 mg/kg q6-hours) as early therapeutic strategies. Subsequently, IVIG (2 g/kg) and intravenous methylprednisolone (30 mg/kg/day for two-days and then, 2 mg/kg/day in three-other days) were administrated. The patient survived near four days in PICU and afterthat transferred to infectious ward, and discharged with good condition at day 10. Her neurological examination at the time of discharge and three months later were normal.

## Discussion

There are growing evidence of neurotropic properties of SARS-CoV-2 inducing several presentations of polyneuropathy, encephalitis, meningitis, acute hemorrhagic necrotizing encephalopathy, leukoencephalopathy, acute disseminated encephalitis (ADEM), Guillen barre, transverse myelitis, febrile seizures, convulsions, and some other possibilities [7].

Although neurological complications of COVID-19 present mainly in patients with severe disease, they have also been reported in non-severe cases [8]. The presence of angiotensin-converting enzyme-2 (ACE-2), a key receptor that is required for the cell entry of SARS-CoV-2, in the olfactory nerves, endothelial cells, along with the brain tissue, indicates that the brain may be a potential direct impact of the virus [7]. Nevertheless, trans-synaptic propagation [9] and inflammatory states [7, 10] may promote the host antibodies or lymphocytes which can impact with cross-reactivity mechanisms [7, 11]. Alexopoulos et al. tested the CSF of eight COVID-19 patients for SARS-CoV-2 antibodies. However, in all patients, the CSF was positive for these antibodies and negative for autoimmune encephalitis antibodies and SARS-CoV-2 PCR, consistent with most previous studies [12]. Mohamed Kamal et al. described a 31-year-old COVID-19 patient who presented with acute behavioral changes and severe confusion. The CSF analysis was consistent with COVID-19 encephalitis, as well as his brain imaging. The SARS-CoV-2 RNA PCR was positive at the time of admission and was not detected two weeks later [13].

Due to the absence of CSF pleocytosis, the suspicious of encephalitis should not be left out [14]. Both of our patients did not undergo brain MRI. Therefore, this remains unclear whether our patients had a mild not proven encephalitis or they experienced their neurological conditions under a non-specific neurological diagnosis. Based on a meta-analysis of data obtained from 193 COVID-19 patients who had an brain and/or spinal MRI and CSF testing for work up, Lewis et al. found that the presence of brain hyper-intense signals or leptomeningeal lesions were associated with increased likelihood of a positive SARS-CoV-2 PCR in CSF. However, they concluded that a positive CSF SARS-CoV-2 PCR is uncommon in these patients, suggesting they are often related to other etiologies like infections, hypoxia, ischemia or metabolic states [15]. Similarly, in another study, among 58 COVID-19 patients, four of them had a positive CSF SARS-CoV-2 RT-PCR results [16]. There is emerging evidence regarding the detection of SARS-CoV-2 RT-PCR in the CSF of pediatric patients affected with COVID-19. Cheraghali et al. reported a 34-month-old child who presented with fever and seizure compatible with the diagnosis of encephalitis. Both nasopharyngeal and CSF SARS-CoV-2 RT-PCR tests were positive [17]. The detection of SARS-CoV-2 RNA in the CSF was also described in a 12-year-old boy who presented with focal encephalitis. The CSF analysis was performed on the fourth day of illness, revealing no pleocytosis, and normal protein and glucose levels. The RT-PCR testing of CSF for SARS-CoV-2 was positive [18]. Considering the scattered reports in this regard, in two literature review by Carrol et al. and Siracusa et al. only six and two patients with neurological manifestations had positive SARS-CoV-2 PCR in their CSF, respectively [19, 20].

Because the CSF SARS-CoV-2 PCR is impacted by several factors such as rapid CSF clearance, contamination from shed airborne virus or blood, low titers, and pre-analytical errors [15, 21, 22], the results may not always be correct. There is the probability of false positive results for SARS-CoV-2 PCR testing in patients with acellular CSF [18]. The absence of pleocytosis, especially acellular CSF is atypical in the setting of viral encephalitis. Our CSF sampling was performed in the acute phase of the illness, just like other reports [17, 18]. However, further studies are needed to determine the time frame of positivity and clearance.

This report is one of the first pediatric reports of positive SARS-CoV-2 PCR in the CSF of two patients with severe COVID-19. Both patients had at least two organ system involvement along with the presence of SARS-CoV-2 infection, compatible with the diagnosis of severe COVID-19 or multisystemic inflammatory syndrome of childhood (MIS-C). Aside from being a novel report, it is important in two other ways. First, the SARS-CoV-2 footprints on CNS, and the second, finding the virus in MIS-C, which thought previously to be a delayed immunity effect. However, on the view of our findings and discussed articles, systematic lumbar puncture seems necessary in patients with neurological manifestations of COVID-19. These observations highlight the need for future studies of CSF in patients with neurological manifestations of COVID-19 for evaluating of SARS-CoV-2 PCR, antibodies, and inflammatory factors.

## Data Availability

All data generated or analyzed during this study are included in this published article.

## Abbreviations

COVID-19: Coronavirus disease 2019
CSF: Cerebrospinal fluid
PCR: Polymerase chain reaction
SARS-CoV-2: Severe acute respiratory syndrome coronavirus disease 2019
ICU: Intensive care unit
MIS-C: Multisystem inflammatory syndrome
CNS and PNS: Central and peripheral nervous systems
PICU: Patient transferred to pediatric ICU
T: Temperature
RR: Respiratory rate
PR: Pulse rate
WBC: White blood cell
ANC: Absolute Neutrophil Count
ALC: Absolute Lymphocyte Count
ESR: Erythrocyte Sediment Rate
CRP: C-reactive protein
BNP: Brain natriuretic peptide
LP: Lumbar puncture
CXR: Chest X-Ray
RT-PCR: Real time-PCR
MRI: Magnetic resonance imaging
IVIG: Intravenous immunoglobulin
ADEM: Acute disseminated encephalitis
ACE-2: Angiotensin-converting enzyme-2
